# Organic compatible solutes of halotolerant and halophilic microorganisms

**DOI:** 10.1186/1746-1448-1-5

**Published:** 2005-08-04

**Authors:** Mary F Roberts

**Affiliations:** 1 Merkert Chemistry Center, Boston College, 2609 Beacon Street, Chestnut Hill, MA 02465, USA

## Abstract

Microorganisms that adapt to moderate and high salt environments use a variety of solutes, organic and inorganic, to counter external osmotic pressure. The organic solutes can be zwitterionic, noncharged, or anionic (along with an inorganic cation such as K^+^). The range of solutes, their diverse biosynthetic pathways, and physical properties of the solutes that effect molecular stability are reviewed.

## 

A dynamic and important property of cells is their ability to rapidly adapt to changes in external media, for example, increasing NaCl. To adjust to increased external NaCl, cells in all three kingdoms accumulate a variety of small molecules in the cytoplasm to counteract the external osmotic pressure. Inorganic cations (K^+ ^and in some cells Na^+^) are often key players in osmotic balance and the osmotic response. However, the diverse collection of organic solutes that organisms accumulate in response to salt stress (also termed osmolytes) is particularly intriguing. Cataloging the occurrence of different molecules and understanding their biosynthesis and regulation by external osmotic pressure have been active areas of research. Studies show that the accumulation of solutes has another role along with osmotic balance. Many osmolytes have been shown to increase the stability of proteins. They appear to act as chemical chaperones in cells, and the mechanism of this stabilization can provide insights into protein folding. The thermostabilizing role of osmolytes has also been exploited for various biotechnology purposes. The aim of this review is an examination of the chemical and biological scope of osmolytes in a wide range of halotolerant and halophilic organisms with an overview of experiments that address why these types of solutes have been naturally selected for osmotic balance. Also included is a brief summary of the biotechnological uses of these organic osmolytes.

The types of organic molecules used for osmotic balance include polyols and derivatives, sugars and derivatives, amino acids and derivatives, betaines, and ectoines and occasionally peptides suitably altered to remove charges [1]. As a general rule of thumb, bacteria and eukaryotes usually accumulate neutral compatible solutes whereas archaea prefer negatively charged solutes [2,3]. Interestingly, archaea tend to modify many of the same neutral or zwitterionic solutes accumulated by eukaryotes or bacteria to make them negatively charged. Osmolytes can either be synthesized by the cell or transported into the cell from the medium. A key feature of these molecules is that they do not inhibit overall cellular functions, although they may modulate individual enzyme activities. This behavior led to labeling them as 'compatible solutes' [4]. Their accumulation helps to maintain turgor pressure, cell volume, and concentration of electrolytes – all important elements for cell proliferation. It is thought that initial events that trigger osmolyte accumulation could include ion channels or other transmembrane proteins sensing differences in external and internal salt concentration, cell volume changes, and/or turgor pressure changes. However, except for transporters, how these physical changes are translated to increased osmolyte synthesis is not known.

## Identification of Osmolytes

Although osmolytes tend to occur at high intracellular concentrations, they do not have unique chromophores and were not considered in much detail and in most cases even identified until high resolution NMR spectroscopy became a routine analytical method. From the 1970s onward, a variety of NMR approaches have been used to identify the organic solutes accumulated by halotolerant and halophilic organisms. Early natural abundance ^13^C NMR studies of cell extracts identified novel solutes, such as ectoine [5], several β-amino acids [6-8], and di-*myo*-inositol-1,1'-phosphate (DIP), the last associated with hyperthermophiles [9,10]. More recent methods using ^1^H NMR and two-dimensional experiments have significantly increased the sensitivity of solute detection [11]. ^1^H NMR methodology can also be used to detect and quantify osmolytes in cell cultures without extraction [12]. Other analytical methods such as HPLC have been used, often to quantify specific solutes as long as an appropriate detection method is available. Refractive index detection is the most general [13], but specific classes of molecules can be derivatized for rapid and sensitive detection (e.g., chromophores added to solutes containing free amino groups [14]). More recent advances have improved on the sensitivity of these other assays. For example, the combination of anion-exchange chromatography and pulse amperometric detection is a very sensitive method that can detect osmolytes such as ectoine after hydrolytic cleavage of the pyrimidine ring [15]. The methodology is sufficiently sensitive that it can be used to screen colonies on agar for solutes.

**Figure 1:**
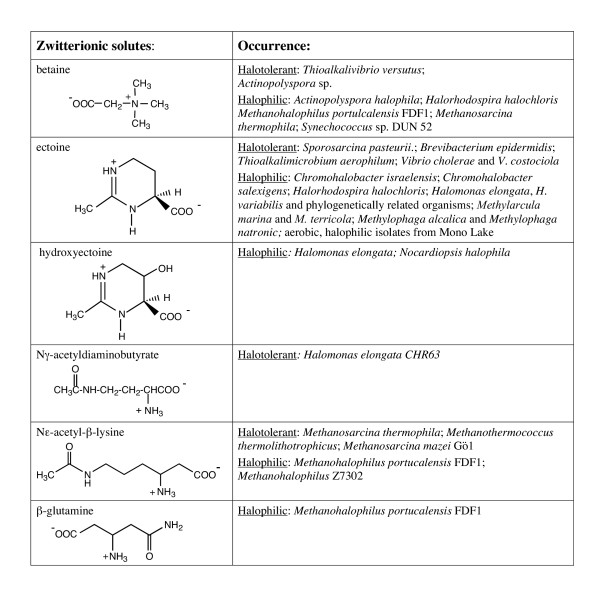
Zwitterionic organic osmolytes detected in bacteria and archaea.

**Figure 2:**
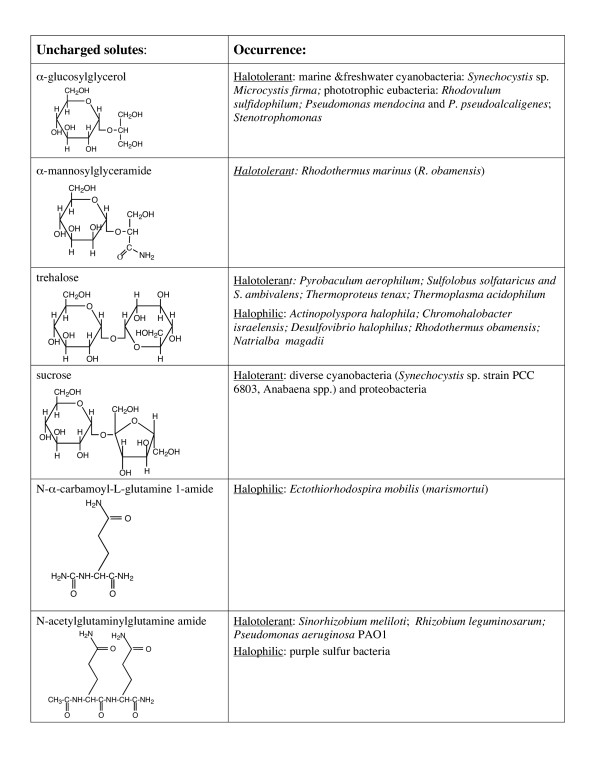
Uncharged organic osmolytes detected in bacteria and archaea.

**Figure 3:**
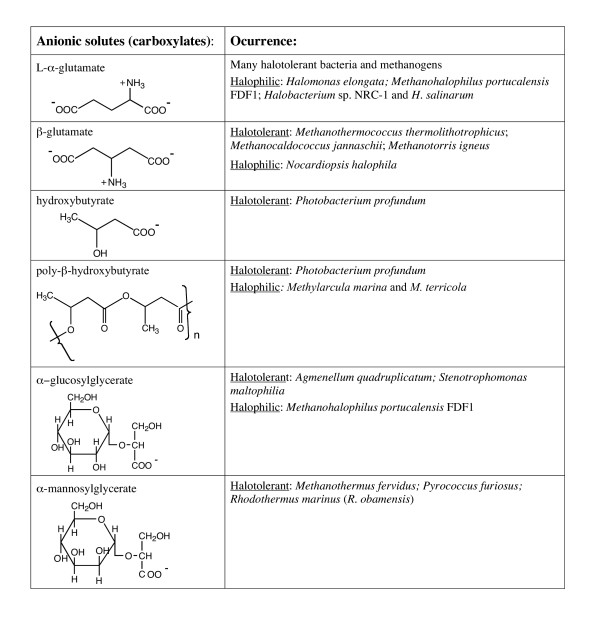
Anionic organic osmolytes containing carboxylates that have been detected in bacteria and archaea.

**Figure 4:**
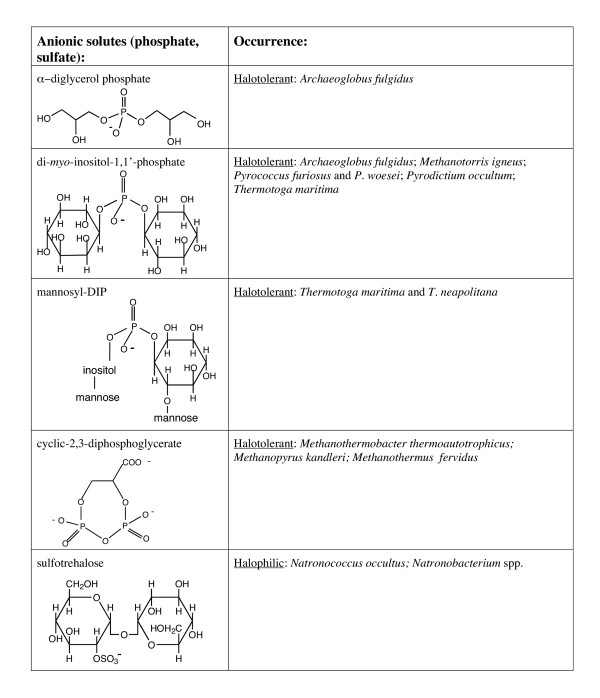
Anionic organic osmolytes containing phosphate or sulfate moieties that have been detected in bacteria and archaea.

Organic osmolytes fall into three general chemical categories: (i) zwitterionic solutes, (ii) noncharged solutes, and (iii) anionic solutes. Structures of these molecules and their occurrence in halotolerant and halophilic microorganisms are presented in Figures 1,2,3,4. Intertwined with these organic solutes are K^+ ^and Na^+ ^which also contribute to osmotic balance in cells.

### A. Zwitterionic Solutes

Free polar amino acids in cells might be expected to play a role in osmotic balance. However, neutral amino acids are not accumulated to high concentrations, presumably because they are intermediates in protein biosynthesis. High and varying concentrations of these compounds could affect diverse cell pathways. Instead, many bacterial and archaeal cells synthesize and accumulate a few zwitterionic molecules derived from amino acids as compatible solutes. Structures of these solutes and where they are found are presented in Figure 1.

#### 1. Betaine

This ubiquitous solute, glycine with the primary amine methylated to form a quaternary amine, is found in halophilic bacteria of diverse phylogenetic affiliation [16]. In most cells where it is accumulated as an osmolyte, the betaine is actively transported from complex medium. Betaine concentrations vary with external NaCl. For example, Imhoff and Rodriguez-Valera [16] showed that for the eight halophiles examined, the average betaine concentrations in 3, 10 and 20% NaCl (0.51, 1.7 and 3.4 M) were 0.21 ± 0.2, 0.65 ± 0.06, and 0.97 ± 0.09 M. A number of methanogens have also been observed to accumulate betaine when grown in rich medium [17]. In contrast to the large number of bacteria that transport betaine into the cell for use as an osmolyte, there are only a few bacteria (e.g., *Actinopolyspora halophila *and *Halomonas elongata*) and one methanogen (*Methanohalophilus portucalensis *FDF1) that are able to synthesize betaine either by oxidation of choline or methylate of glycine [18-20].

#### 2. Ectoine and hydroxyectoine

Ectoine, a cyclic tetrahydropyrimidine (1,4,5,6-tetrahydro-2-methyl-4-pyrimidinecarboxylic acid) can almost be considered a marker for halophilic bacteria. As shown in Figure 1, it is synthesized by a wide range of bacteria, both halotolerant and halophilic varieties. This solute was first detected in the halophilic, phototrophic *Halorhodospora halochloris *[5]. The intracellular ectoine concentration was shown to increase with increased extracellular NaCl. Screens of a number of microorganisms have shown that ectoine is the major osmolyte in aerobic chemoheterotrophic bacteria [1]. It is also the major solute in bacterial strains isolated from alkaline, hypersaline Mono Lake [21]. More recently it has also been observed in the moderately halophilic methylotrophic bacteria *Methylarcula marina*, *M. terricola*, and *Methylophaga *sp. [22,23]. A variant of this solute, hydroxyectoine, has been detected in halotolerant *Sporosarcina pasteurii *grown in high osmolarity medium [24].

Growth conditions have been shown to affect the intracellular ectoine pool. For example, in halotolerant *Brevibacterium *sp., the size of the intracellular ectoine pool depends not only on the external salt concentration but on the type of carbon source and aeration level [25]. Ectoine accumulation can also depend on growth stage. In *Chromohalobacter israelensis *(formerly *Bacterium *Ba1), ectoine only accumulated when the cells were grown in greater than 0.6 M NaCl, and only in exponentially growing cells [26]. Some microorganisms, e.g., *Brevibacterium epidermidis*, can also metabolize ectoine [27], perhaps a useful trait if external salt concentrations decrease. In that halotolerant organism, ectoine accumulation occurs only with salt stress, not sugar stress.

The ability to accumulate ectoine can give an organism an ecological advantage. High-osmolarity-adapted *Vibrio cholerae *cells accumulate ectoine and betaine and out grow non-adapted cells [28]. This has implications for *V. cholerae *population dynamics when seawater and freshwater and their attendant microbes mix.

#### 3. Nε-acetyl-β-lysine and β-glutamine

Methanogens have a notably different strategy than many other organisms in that they accumulate several β-amino acids for osmotic balance. These solutes provide an excellent strategy for producing a compatible solute since β-amino acids are not incorporated into proteins or other macromolecules. At high external NaCl (>1 M), two zwitterionic β-amino acids have been shown to accumulate in response to external NaCl. Nε-acetyl-β-lysine has been detected in a wide range of mesophilic and a few thermophilic methanogens [7,29-31]. β-Glutamine has been detected in *Methanohalophilus *species where it is synthesized and accumulated along with Nε-acetyl-β-lysine and betaine [8]. ^13^C-pulse/^12^C-chase and ^15^N-pulse/^14^N-dilution NMR experiments can be carried out where cells are grown in the presence of an NMR-active isotope (typically ^13^CO_2 _or ^15^NH_4_Cl) for some time. The labeled compound is then removed or significantly diluted with unlabeled material (^12^CO_2 _or ^14^NH_4_Cl). Loss of the NMR-active isotope then monitors the turnover of these β-amino acid solute pools in the cells. Both Nε-acetyl-β-lysine and β-glutamine exhibit little if any turnover in cells as expected if these are used only for osmotic balance [30,31].

### B. Noncharged solutes

Few molecules that are polar but lack any formal charges have been identified as osmolytes in halophilic bacteria, although they are well represented in eukaryotes. For example, glycerol is prevalent as an osmolyte in marine and halophilic *Dunaliella *[32,33]. Glycerol accumulation is also a characteristic of halotolerant yeast *Debaryomyces hansenii *as well as the black yeast *Hortea werneckii*, and adaptation of this eukaryotic organism to high NaCl requires glycerol accumulation [33]. *Myo*-inositol, another polyol, is used as an osmolyte in several eukaryotes. Neither of these polar noncharged solutes has been identified as an osmolyte in bacteria or archaea (or associated with halophiles). However, negatively charged derivatives of both glycerol and inositol are accumulated by archaea. The few uncharged solutes that are used by halotolerant bacteria and archaea include several carbohydrates and an amino acid/dipeptide modified to neutralize all charged groups (Figure 2).

#### 1. Carbohydrates

Few carbohydrates are used for osmotic balance, perhaps because those with a reducing end are chemically reactive, and in a sea of proteins these noncharged solutes would be likely to react with surface amino groups. To avoid this, the reactive end of the sugar forms a glycosidic bond with a small neutral molecule, either glycerol or glyceramide. The neutral derivatized sugars glucosylglycerol and α-mannosylglyceramide [34] have been detected in a few bacteria (Figure 2). α-Glucosylglycerol is accumulated by a member of the Proteobacteria, *Stenotrophomonas *[35]. This organism has a large number of potential biotechnology uses (many based on its ability to use uncommon carbon sources), one of which is the production of glucosylglycerol. α-Mannosylglyceramide is accumulated in *Rhodothermus marinus *[34].

The non-reducing glucose disaccharide trehalose is used by organisms to counteract drying, but it also serves as an osmolyte. In *Actinopolyspora halophila *trehalose represents 15% w/v in cells grown in 24% w/v NaCl [36]. However, in some cells, its accumulation is preferred at lower NaCl. For examples, in *Chromohalobacter israelensis*, trehalose is only an important solute when the cells are grown with <0.6 M external NaCl [26]. In the sulfate-reducing bacterium *Desulfovibrio halophilus*, trehalose is the major osmolyte. When grown in 15% (2.5 M) NaCl in the absence of a source of betaine, the cells accumulated 8 μmol trehalose/mg protein and ~2.5 μmol K^+^/mg protein [37].

Disaccharides without modifications, notably sucrose, can be transported by some halotolerant and halophilic organisms, and this can enhance growth in higher NaCl. Sucrose is synthesized in cyanobacteria and proteobacteria [38,39] where it is usually associated with lower salt tolerance strains. *Synechocystis *sp. strain PCC 6803 tolerates up to 1.2 M NaCl. In those cells, the sucrose is a minor solute (glucosylglycerol is the major osmolyte). However, the sucrose is critical for stationary phase survival under salt stress conditions [40]. This observation was rationalized by proposing that the sucrose could regulate metabolic pathways that are active under the nutritional stress conditions of stationary phase [40].

While it is rarely synthesized in bacteria, sucrose is a major osmoprotectant in plants, and synthesis of sucrose is similar in both cyanobacteria and plants. There are two distinct pathways. In freshwater and marine cyanobacteria, sucrose is synthesized synthesized from fructose-6-phosphate and a sugar nucleotide (UDP-glucose) in two steps using sucrose phosphate synthase and sucrose-phosphate phosphatase [41]. Filamentous cyanobacteria (e.g., *Anabaena *sp.) use a different pathway, sucrose synthase, which reversibly converts fructose and ADP-glucose (or UDP-glucose) to sucrose [42]. Since sucrose-synthesizing enzymes cannot be identified in other bacteria or archaea, it is thought that sucrose synthesis in eukaryotes was acquired by endosymbiotic cyanobacteria that were the ancestors of chloroplasts [43].

#### 2. Uncharged Amino Acids and Peptides

Two solutes in this class have been identified as osmolytes: (i) a carboxamine, and (ii) an acetylated neutral glutamine dipeptide. In both solutes, modifications mask the charged α-amino and α-carboxyl groups. N-α-Carbamoyl-L-glutamine 1-amide, an unusual amino acid derivative, is accumulated by halophilic phototrophic bacterium *Ectothiorhodospira marismortui *(also known as *Ectothiorhodospira mobilis*) [44]. The dipeptide N-acetylglutaminylglutamine amide is synthesized by several halophilic purple sulfur bacteria [45,46].

### C. Organic anions

Cells have a negative potential inside and often quite high intracellular K^+^. Negatively charged solutes could serve to balance high intracellular K^+ ^as well as counteract osmotic pressure. Indeed, at lower external NaCl, many bacteria (including *H. elongata *which also synthesizes ectoine) and archaea use L-α-glutamate as an osmolyte. In methanogens, high NaCl often causes the cells to switch from anionic glutamate isomers to the zwitterionic solute Nε-acetyl-β-lysine for osmotic balance [29,31]. Anionic solutes used by bacteria and archaea for osmotic balance can have a carboxylate supply the negative charge (Table 3) or contain phosphate or sufate groups (Figure 4).

#### 1. β-Glutamate

Methanogens tend to accumulate β-glutamate as well as α-glutamate for osmotic balance. ^13^C-pulse/^12^C-chase NMR experiments that monitor solute turnover for α- and β-glutamate have shown that the α-glutamate pool is metabolized and replenished while the β-amino acid pool is relatively static, hence it is an ideal compatible solute [30,31,47,48]. In *Methanothermococcus thermolithotrophicu*s, both the α- and β-glutamate levels increase with increasing external NaCl [49]. However, there appears to be a threshold for the glutamates in this organism. The negatively charged glutamates are accumulated when the external NaCl is less than 1 M. In that regime, the total intracellular glutamates occur at concentrations comparable to the intracellular K^+^. Above 1 M NaCl, zwitterionic Nε-acetyl-β-lysine becomes the major solute [31,50]. The accumulation of the zwitterions at high NaCl could indicate that it is now energetically too costly to increase K^+ ^and hence the anionic glutamates that aid in neutralizing much of the K^+ ^are not needed. In support of this are observations in *M. thermolithotrophicus *that within 30 min of switching cells from 0.67 to 1.4 M NaCl, both K^+ ^and glutamate concentrations increase transiently then later decrease as the zwitterion is eventually synthesized [48].

While most studies identifying β-glutamate have concentrated on methanogens, this solute has been detected in a few bacteria as well. For example, it has been detected in the Gram-positive organism *Nocardiopsis halophila*, which also accumulates the zwitterionic hydroxyectoine [51].

#### 2. β-Hydroxybutyrate and derivatives

Soluble poly-β-hydroxybutyrates, normally used as carbon reservoirs in cells, have been detected in moderate concentrations in a number of organisms, including *Methylarcula marina *and *Methylarcula terricola *[22] and in the deep sea organism *Photobacterium profundum *SS9 [52]. The role of polyhydroxybutyrates in the deep sea bacterium is particularly intriguing. In *P. profundum*, betaine and glutamate represent the major solutes when the cells are grown at 1 atm. However, when grown at 280 atm, β-hydroxybutyrate and polymers of this solute accumulate and become the major solutes. At a fixed hydrostatic pressure, β-hydroxybutyrates also increase with increasing external NaCl (particular at high pressures), indicating that the monomer and possibly the polymer (although the enhanced intensity in the NMR resonances for this compound could also indicate increased chain length) function as conventional osmolytes. Because their intracellular levels respond to hydrostatic as well as osmotic pressure, these β-hydroxybutyrate solutes in this organism have been termed 'piezolytes' [52].

#### 3. Anionic polyols and carbohydrates

In bacteria, high intracellular concentrations of negatively charged carbohydrates are not very common. Two such solutes that have been detected include α-glucosylglycerate and α-mannosylglycerate. These solutes tie up the reactive end of the sugar in a glycosidic bond with a hydroxyl group of glyceric acid. α-Mannosylglycerate, accumulated by several *Rhodothermus *spp., is higher in exponential phase cells and decreases abruptly as cells enter stationary phase [34]. These cells accumulate both the anion mannosylglycerate and the neutral α-mannosylglyceramide. Which of these two solutes dominates depends on stress conditions. Under temperature stress of the cells, *R. marinus *is biased towards accumulating mannosylglycerate; increased NaCl favored accumulation of the neutral α-mannosylglyceramide rather than the organic anion [34]. Glucosylglycerate has also been observed in *Methanohalophilus portucalensis *when those cells are grown with methanol rather than methylamine as the substrate for methanogenesis [30]. It is a relatively minor contributor to osmotic balance under those conditions, but the cells do synthesize it. Its turnover, measured by NMR, is roughly twice as slow as α-glutamate and 2–4 times faster than turnover of the zwitterions betaine and Nε-acetyl-β-lysine [30].

Halotolerant archaea (excluding most methanogens) tend to accumulate organic anions where the negative charge is often provided by a phosphate moiety and in some cases by sulfate added to a noncharged solute (Figure 4). Representatives of this class of compounds include the glycerol derivative α-diglycerol phosphate [53] and a series of *myo*-inositol phosphodiesters based on di-*myo*-inositol-1,1'-phosphate (DIP) [9,10]. Phosphodiesters are better choices than phosphomonoesters for accumulation at high concentrations since they will have weaker interactions with cations (particularly divalent cations). DIP and its relatives (e.g., mannosyl-DIP [54]) are associated with halotolerant hyperthermophiles. The intracellular concentration of these solutes increases with external NaCl, but the increase is usually more striking with growth temperatures above 80°C [10,55-57]. In *Archaeoglobus fulgidus *grown at 76°C, α-diglycerol phosphate is the major osmolyte, varying with external NaCl; little if any DIP is detected. However, at 87°C DIP concentrations are comparable to the α-diglycerol phosphate [58]. The association of DIP with very high temperatures suggests that this and related solutes have a role in stabilizing macromolecules to high temperature, although why this odd sugar is used is not clear. Synthesis of DIP from glucose-6-phosphate requires significant energy, so that there must be a reason for its accumulation.

A few archaea have been seen to accumulate cyclic-2,3-diphosphoglycerate, an unusual cyclic pyrophosphate with a net -3 charge. This solute was first detected in strains of *Methanothermobacter thermoautotrophicus *[59,60], a thermophilic methanogen that is usually grown in medium containing low NaCl. In the Marburg strain of that organism 1,3,4,6-tetracarboxyhexane, a component of methanofuran, is also a major solute [61]. Cyclic-2,3-diphosphoglycerate is also accumulated in *Methanobrevibacter smithii*, *Methanopyrus kandleri *and *Methanothermus fervidus *[58,62]. However, at least in *M. thermoautotrophicus *it has unusual behavior compared to most osmolytes. It exhibits very rapid turnover in the cells (compared to the cell doubling time) and appears to be fixed into a polymer pool from which it can be retrieved in times of stress [63,64]. Furthermore, its intracellular concentration does not vary much, even when the cells are grown in 0.4 NaCl [61]. This behavior could suggest it has a primary role as a carbon and phosphate storage compound in these methanogens.

Another unusual anionic solute found in haloalkaliphilic archaea is sulfotrehalose [65]. This derivative of trehalose with a sulfate at C2 of one of the glucose rings was the major solute in several *Natronococcus *and *Natronobacterium *species grown in defined media. The intracellular sulfotrehalose increases with increased external NaCl and is accumulated in amounts comparable to the intracellular K^+ ^(P. Jablonski, unpublished results). However, the sulfotrehalose can be replaced by sucrose, in which case the cells have roughly double the amount of organic solute. Interestingly, sulfatides with sulfotrehalose (modified tehalose 2'-sulfate with acyl chains on the other glucose moiety attached to C2 and C3) are synthesized in *Mycobacterium tuberculosis *[66]. Whether or not such sulfatides exist in the archaea has not been determined.

### D. K^+ ^and other inorganic ions

The high concentration of organic anions in many halophiles requires counterions such as K^+ ^and/or Na^+^. However, there are halophiles that exclusively use inorganic ions for osmotic balance. Halophilic aerobic archaea have been shown to have high K^+ ^and Cl^- ^[67], although absolute amounts appear to depend dramatically on the method of analysis and/or growth conditions. There are also bacteria with exceedingly high intracellular K^+^. In *Salinibacter ruber*, an obligatory aerobic chemoorganitrophic and very halophilic bacterium, K^+ ^is the major intracellular component of osmotic balance with 11–15 μmol K^+^/mg protein [68]. Organic solutes are relatively low in this organism; these studies used elemental analysis with EM and an X-ray spectrum to describe constituents and flame-photometric determination of K^+^. *S. ruber *occupies a relatively unique position in the bacterial kingdom. Its closest relative, *Rhodothermus marinus*, uses α-mannosylglycerate and α-mannosylglyceramide as osmolytes for osmotic balance [34].

Other microorganisms that do not appear to use organic osmolytes for balance include anaerobic fementative members of the families Halobacteroidaceae and Halanaerobiaceae [67-70]. *Halobacterium salinarum*, an archaeon, has been reported to accumulate 12 μmol K^+^/mg protein. While some organic solutes were observed (≤50 mM), at those low concentrations they are unlikely to play a major role in osmotic balance, although they may aid in charge balance within the cells. Using a cell volume of 2.75 μl/mg proteins for *H. salinarum*, the estimated intracellular K^+ ^is 4.4–4.8 M comparable to the Na^+ ^concentration in the medium. Extracting cell pellets prior to K^+ ^analyses led to considerably lower values (~1 M for *S. ruber *and ~3 M for *H. salinarum*) for K^+^, suggesting ion leakage. Intracellular Na^+ ^was also high in the pellet extracts, which could suggest problems with this type of analysis. Alternatively, the difference might reflect complexed versus uncomplexed ions. Halophilic sulfate-reducing bacteria, e.g., *Desulfohalobium retbaense *and *Desulfovibrio halophilus*, like the haloarchea, appear to use inorganic salts for osmotic balance.

In contrast to the archaeal halophiles, many of the halophilic bacteria do not have exceptional high K^+^. For example, *Halomonas elongata *accumulates 1.1 μmol K^+^/mg protein when grow with yeast extract and 2.2 μmol/mg protein when grown in defined medium with glucose as the sole carbon source. In *Halanaerobium acetethylicum *grown in medium with 1.7 M NaCl, the internal cytoplasmic Na^+ ^and Cl^- ^are 0.92 and 1.2 M, respectively, while K^+ ^and Mg^2+ ^concentrations in cells are 0.24 and 0.02 M, respectively [70].

Although K^+ ^(and occasionally Na^+^) appears to be the major intracellular cation, there are reports that, in some cells, Mg^2+ ^can reach moderately high concentrations. Heldal and coworkers [71] have found high Mg^2+ ^(close to 0.9 M) in native marine bacteria under conditions of low dissolved organic carbon. The intracellular Mg^2+ ^was dramatically reduced (<0.2 M) when nutrient levels increased. They suggested that high intracellular Mg^2+ ^is a marker of carbon limitation.

Chloride is the most prevalent inorganic anion in halophiles that do not accumulate organic anions. Molar concentrations of chloride have been detected in several halophilic archaea. This anion is pumped into cells by halorhodopsin or cotransported with Na^+^. While this anion certainly can contribute to osmotic balance, it appears to have more critical roles in haloadaptation [72]. For example, chloride has been shown to regulate betaine transport. Aside from chloride, little is known about the inorganic anion composition of halophiles. However, a recent FT-IR study of intact bacteria during growth indicates that in *H. salinarum *and *Halococcus morrhuae*, large changes occur in the concentration of sulfate ion in the cells [73]. Maximum sulfate occurs during the mid-part of the exponential phase.

### E. Cocktails of organic solutes

One of the things arising from the studies of different halotolerant and halophilic organisms is that most cells use an array of solutes, not a single one, for osmotic balance. When a single solute is detected it is often supplied by the medium and efficiently transported into the cell. However, left to its own device, the typical bacterial or archaeal cell synthesizes several molecules that together contribute to osmotic balance. Sometimes this is a combination of anions and zwitterions, but often several solutes with the same net charge. Archaea provide particularly intriguing examples of this strategy, although an explanation for the diversity of solutes in a given organism is lacking.

*Methanothermococcus thermolithotrophicus *accumulates the anionic α- and β-glutamate when grown in medium with less than 1 M NaCl [49]. Cells adapted to higher external NaCl concentrations switch to accumulating a zwitterions, Nε-acetyl-β-lysine [31,48,50]. Since the glutamate concentration is roughly the same as intracellular K^+^, the switch to accumulating the zwitterions could be the result of an impaired K^+ ^pump.

*Methanohalophilus portucalensis*, a halophilic methanogen, accumulates three zwitterions over its growth range: betaine, Nε-acetyl-β-lysine, and β-glutamine [8]. α-Glutamate is detected, but its intracellular concentration is relatively low and does not increase with increased external NaCl. Of the three zwitterions, β-glutamine is only accumulated to large amounts at the high NaCl end of the growth range. Several conditions can affect the balance among these three zwitterions [30]. The cells can grow on trimethylamine or methanol as the substrate for methanogensis, and the substrate with nitrogen promoted accumulation of the two solutes containing two nitrogen atoms (Nε-acetyl-β-lysine, and β-glutamine). Supplying precursors of these solutes (glycine, lysine, glutamate) has little effect on the distribution of the three zwitterions. Betaine is the only solute that could suppress synthesis of both Nε-acetyl-β-lysine and β-glutamine when it is present in the medium [74].

Balancing several different anionic osmolytes has also been observed in the halotolerant, hyperthermophilic *Methanotorris igneus*, which accumulates L-α-glutamate, β-glutamate, and DIP [10]. Increased external NaCl leads to preferential increases in the intracellular β-amino acid; thermal stress causes increases in DIP levels. Multiple anionic solutes are also accumulated in other hyperthermophilic archaea. In *Archaeoglobus fulgidus*, glutamate, DIP and α-diglycerol phosphate are used for osmotic balance [53]. In these cells, it is the α-diglycerol phosphate that was most sensitive to external NaCl while heat enhanced DIP synthesis and accumulation.

## Biosynthesis of Osmolytes – Novel Pathways and Regulation

### A. Betaine

**Figure 5:**
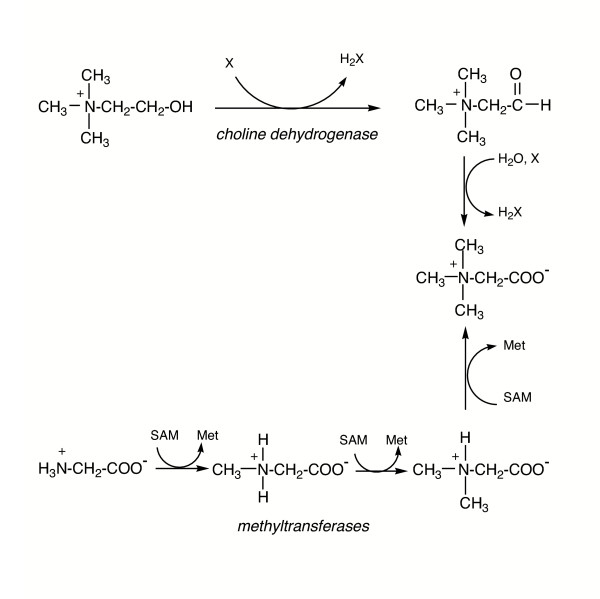
Pathways for synthesizing betaine in bacteria and archaea.

Microorganisms have two different general pathways for synthesizing betaine (Figure 5). The oxidative pathway can occur with a single soluble enzyme (choline oxidase in Gram-positive soil bacteria [75]) or require two distinct soluble enzymes (choline monooxygenase and betaine-aldehyde dehydrogenase in higher plants [76]), or it can occur with a membrane-associated system coded by four genes in the bet operon (in marine invertebrates and bacteria including *Escherichia coli*). The last system has been studied genetically [77], with genes identified for choline dehydrogenase (*betA*), betaine-aldehyde dehydrogenase (*betB*), a choline transporter (*betT*) and a putative regulator (*betI*). The choline dehydrogenase catalyzes oxidation of choline to betaine aldehyde, which is then oxidized to betaine by the *betB *gene product. In *Pseudomonas*, an electron acceptor other than O_2 _is used for choline oxidation with suggestions that PQQ is the acceptor [78]. In *Actinopolyspora halophila*, choline is oxidized to betaine aldehyde then to betaine [36]. The aldehyde is produced with O_2 _consumption and H_2_O_2 _generation. The final oxidation to betaine uses reduction of NADP^+^.

Even organisms that do not accumulate betaine in response to osmotic stress may have homologues of the genes for synthesizing this solute. *Halomonas elongata *does not appear to accumulate betaine. However, the organism does have a gene that codes for the oxidation of choline to betaine [79]. Recently the choline dehydrogenase from that organism was expressed in *E. coli *and characterized. This enzyme can use O_2 _if no other electron acceptors are available, although V_max _decreases four-fold compared to kinetics with an acceptor such as phenazine methosulfate. Both choline and the betaine-aldehyde are converted to betaine. Although a glycine box suggestive of FAD^+ ^as a cofactor was seen in the sequence, there is no experimental evidence for FAD^+ ^as a cofactor. These observations prompt two questions: (1) what cofactor is used by this choline dehydrogenase, and (ii) under what conditions is this gene expressed?

Several microorganisms can also generate betaine by successively methylating glycine. GSMT (glycine sarcosine methyltransferase) and SDMT (sarcosine dimethylglycine methyltransferase) in *Halorhodospira halochloris *and *Actinopolyspora halophila *transfer the methyl group of S-adenosylmethionine to two different types of amines [18,36,80]. Betaine synthesis from glycine in a halotolerant photosynthetic organism *Aphanothece halophytica *was also carried out by GSMT and DNMT activities [81]. Only one methanogen, *Methanohalophilus portucalensis*, has been shown to synthesize betaine de novo [8]. In these anaerobic cells, ^13^C NMR labeling experiments suggest that betaine is generated by successive methylation of glycine [20]. If cells are grown with 10 mM betaine in the medium, accumulation of other osmolytes (Nε-acetyl-β-lysine, β-glutamine and glucosylglycerate) is suppressed [74]. However, supplying cells with betaine precursors does not necessarily alter the distribution of osmolytes. Exogenous glycine or sarcosine had no effect on betaine accumulation. Even though the glycine (^13^C-labeled) was shown to be internalized by the cells [74], betaine synthesis and suppression of other osmolytes only occurs with N, N-dimethylglycine added. This suggests that it is the N, N-dimethylglycine intracellular concentration that regulates betaine synthesis and accumulation. Recently a 240 kDa N-methyl transferase has been isolated and partially characterized (M.-C. Lai, C.-C. Wang, M.-J. Chuang, Y.-C. Wu, and Y.-C. Lee, personal communication). The source of the methyl groups transferred to glycine is (perhaps not surprisingly) S-adenosylmethionine. While mammalian activites usually show very specific methyltransferase activity (e.g., glycine N-methyltransferase), an aggregate of similar mass proteins (but with different pI values) carries out all three methylation activities in the methanogen. Different subunits in the aggregate are optimized for different methyl transfers; the K^+ ^concentration also differentially modulates the methyltransferase activities [[82], M.-C. Lai and coworkers, personal communication]. The last result suggests that betaine accumulation is likely regulated by the internal K^+ ^concentration in these cells.

### B. Ectoine

**Figure 6:**
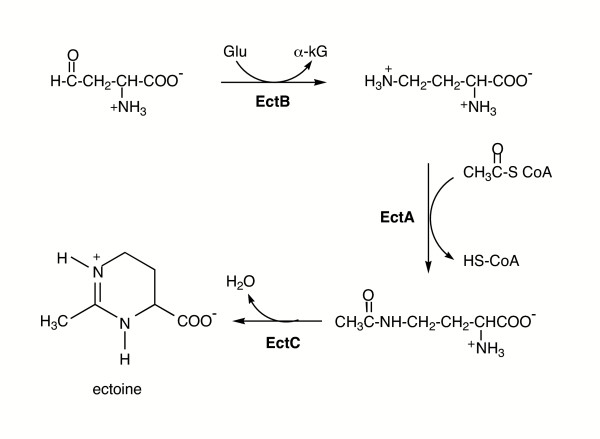
Biosynthetic pathway for ectoine.

The biosynthesis and regulation of ectoine in cells have been studied in several different bacteria, both Gram-negative and Gram-positive. Biosynthesis of ectoine in *H. elongata *has been studied in the greatest detail. The entry molecule into ectoine biosynthesis is aspartate semialdehyde, which is an intermediate in amino acid metabolism [83]. A shown in Figure 6, the aldehyde is converted to L-2,4-diaminobutyric acid, which is then acetylated to from Nγ-acetyldiaminobutyric acid (NADA). The final step is the cyclization of this solute to form ectoine. The genes for biosynthesis of this solute were identified after the isolation of salt-sensitive mutants led to cloning of genes [84]. Ectoine synthesis is carried out by the products of three genes: *ectABC*. The *ectA *gene codes for diaminobutyric acid acetyltransferase; *ectB *codes for the diaminobutyric acid aminotransferase; *ectC *codes for ectoine synthase [19,85]. The three recombinant *H. elongata *Ect enzymes have been characterized. The first enzyme (a 260 kDa complex of 44 kDa subunits) generates the diaminobutyrate by transaminating the aspartate semialdehyde with glutamate. Both pyridoxal 5'-phosphate and K^+ ^are necessary for the diaminobutyrate aminotransferase activity [86]. The aminotransferase of step two is activated by 0.5 M NaCl (and similarly by KCl). The last enzyme involved, ectoine synthase, is also activated by NaCl. This suggests that ectoine accumulation is partially regulated by intracellular cations.

In *Chromohalobacter salexigens*, the *ectABC *genes are regulated at the transcriptional level [87]. Osmoregulated promoters with sequence homology to general stress σ factor have been identified. Since ectoine levels are modulated with betaine present, there must be additional post-transcriptional control. The effect of betaine on *ectABC *expression and ectoine accumulation was also shown in *Marinococcus halophilus *[88]. In defined medium, the intracellular level of ectoine increases with NaCl and suppresses accumulation of trehalose. However, in complex medium, betaine is accumulated and ectoine synthesis is suppressed.

In most organisms, it is thought that hydroxyectoine is synthesized directly from ectoine. However, *H. elongata *has an alternate pathway that was observed in strains defective in EctC. These mutants that can not synthesize ectoine can still convert NADA directly to hydroxyectoine [89]. Canovas et al. [89] proposed that NADA is hydroxylated to 3-hydroxyl-Nγ-acetyldiaminobutyrate, which is then cyclized to hydroxyectoine by 'hydroxyectoine synthase.' The zwitterionic precursor of ectoine can also be accumulated for osmotic balance. NADA has been detected in a salt-sensitive strain of *H. elongata *[89] that grows optimally with 0.75 to 1.0 M NaCl. It accounts for 80% of the organic solute pool for cells grown in 1.5 M NaCl) with ectoine (6%) and hydroxyectoine (12%) also present. NADA confers higher osmotic stability to the cells than in a *H. elongata *mutant where diaminobutyrate accumulates [84]. Thus, this solute, but not its diaminobutyrate precursor (which would have a net positive charge) can act as a compatible solute if ectoine synthesis is blocked.

### C. β-amino acids

Over the past few years, genes have been identified or proteins isolated or cloned that confirm pathways initially proposed based on ^13^C isotopic labeling of these solutes. The pathway originally proposed for biosynthesis of Nε-acteyl-β-lysine has two key enzymes: (i) isomerization of α-lysine to β-lysine catalyzed by a lysine aminomutase, then (ii) acetylation of the ε-amino group [20,31]. Recently the genes coding for these two enzymes were identified in *Methanosarcina mazei *Gö1 [90]: *ablA *codes for the aminomutase while *ablB *codes for the β-lysine acetyltransferase. Expression of the two genes, which are organized in an operon, is salt dependent in *M. mazei*. Several other methanogens, including *Methanococcus maripaludis*, have homologous genes [90]. Deletion of the *abl *operon in *M. maripaludis *generates cells incapable of growth in high salt medium. It will be interesting to characterize the methanogen lysine aminomutase and to compare it to the catabolic enzyme from bacteria that carries out the same chemistry.

**Figure 7:**
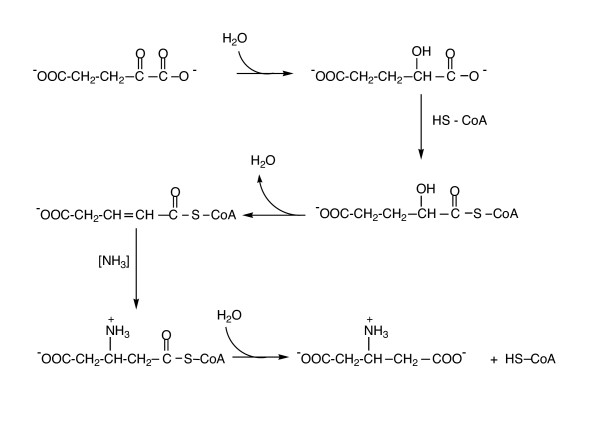
Proposed biosynthetic pathway for β-glutamate.

Early NMR evidence ruled out a glutamate aminomutase activity as a means of generating β-glutamate [31] but did not identify precursors. As shown in Figure 7, the most likely pathway (proposed based on enzyme activities found in *Methanocaldococcus jannaschii *by M. Graupner, H. Xu, and R. H. White, personal communication) starts with the reduction of α-ketoglutarate to α-hydroxyglutarate, which is converted to its coenzyme A ester. Elimination of water from the α-hydroxyglutaryl-CoA generates glutaconyl-CoA, which forms β-glutamyl-CoA when ammonia is added (although the direct source of ammonia is not clear). Hydrolysis of the CoA ester generates β-glutamate. The products of the MJ0800 and MJ0400 genes have been identified as the enzymes responsible for water elimination in this pathway by R. H. White and coworkers (personal communication).

While not all the enzymes for synthesizing β-glutamate have been identified, conversion of β-glutamate to β-glutamine is done in *Methanohalophilus portucalensis *FDF1 by glutamine synthetase [20,91]. That GS has unusual properties compared to other studied GS enzymes. In particular, its activity with β-glutamate as substrate is much higher than that of other organisms [91]. Regulation of the enzyme must occur in the cell, because β-glutamine is only accumulated to NMR-detectable levels in *M. portucalensis *when the cells are grown at higher NaCl [8]. Although the in vitro K_m _values for both α- and β-glutamate in this organism appear quite high, there is likely to be another mechanism responsible for regulation of the synthesis of this solute by glutamine synthetase and accumulation for osmotic balance.

### D. DIP

**Figure 8:**
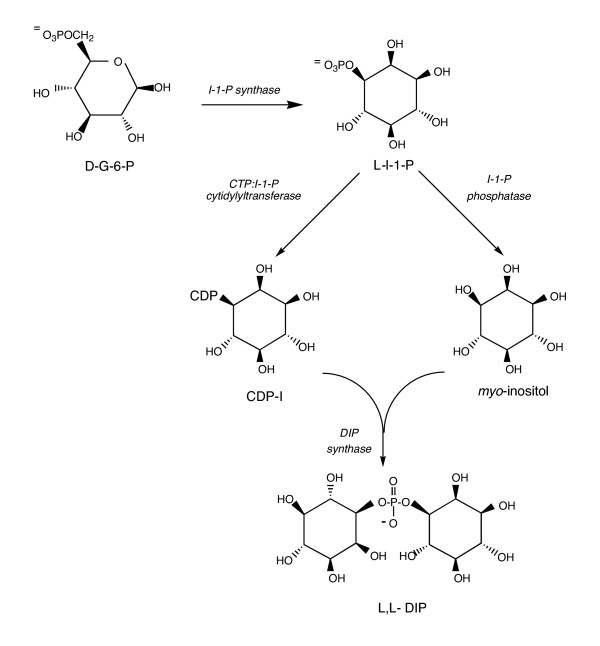
Proposed pathway for DIP biosynthesis in hyperthermophilic organisms.

Data from NMR experiments using ^13^C-labeled precursors to label DIP in *Methanotorris igneus *coupled with in vitro assays with postulated intermediates [92] led to a pathway for the biosynthesis of DIP that includes four steps (Figure 8): (i) conversion of D-glucose-6-phosphate to L-inositol-1-phosphate (L-I-1-P) via inositol-1-phosphate synthase (IPS); (ii) hydrolysis of the L-I-1-P by inositol monophoshatase; (iii) coupling of the L-I-1-P with CTP to form CDP-inositol; and (iv) generation of the phosphodiester linkage by condensing CDP-inositol with L-I-1-P (via a 'DIP synthase' activity for whom there is yet no candidate in genomes of organisms that accumulate DIP). In *P. woesei*, but not in *M. igneus*, DIP could also be generated from incubations of crude cell extracts with GTP and I-1-P [93]. This finding can be explained by the same condensation mechanism, but assuming a multifunctional 'DIP synthase' that catalyzes not only the condensation of CDP-I and *myo*-inositol but the dephosphorylation of I-1-P as well (presumably without releasing the dephosphorylated product, *myo*-inositol).

The IPS reaction has been examined in several hyperthermophiles (*Archaeoglobus fulgidus*, *Methanotorris igneus*, *Pyrococcus furiosus*, *P. woesei*, and *Thermotoga maritima*) known to accumulate this solute (L. Chen and M.F. Roberts, unpublished results). IPS activities in crude extracts are ubiquitous in these organisms and fall into two classes: (i) IPS dependent on divalent cations (Mn^2+ ^or Zn^2+^) is detected in *A. fulgidus*, while (ii) the IPS activities from the other organisms are not activated by metal ions or NH_4 _^+ ^(the cofactor for all other known IPS). Although it is the first step in DIP synthesis, the IPS reaction is unlikely to be the point where DIP synthesis and accumulation are regulated since many archaea incorporate inositol into their lipids. If they incorporate L-I-1-P into lipids, then the second step, the generation of *myo*-inositol could be a way to regulate flow of resources into DIP.

The archaeal IMPase enzymes, easily identified by sequence homology to mammalian IMPases, have unusual properties. They exhibit similar substrate specificity to eukaryotic IMPases with one curious exception – they very specifically can dephosphorylate the phosphate on C1 of fructose bisphosphate [94]. The FBPase activity identifies this class of enzymes as dual phosphatases that can process substrates in completely different pathways. FBPase activity gives it a potential role in gluconeogenesis. However, a 'true' FBPase with a homologue in all archaeal genomes was recently purified and cloned [95,96], so that the IMPase/FBPase in archaea may normally function as an IMPase, (although, *Methanocaldococcus jannaschii *does not accumulate DIP, has no IPS sequence homologue, yet still has a gene for an IMPase/FBPase homologue which has been expressed and characterized [97]). Nonetheless, it is intriguing that an enzyme that could act as either an IMPase or FBPase under the right circumstances (salt or temperature stress?) could link carbohydrate synthesis with responses to stress. At least for the IMPase from *A. fulgidus*, there are hints as to what could regulate this enzyme. This IMPase has two spatially close cysteine residues that can be oxidized to form a disulfide (either by vigorus bubbling with O_2 _at 85°C or by adding oxidized *E. coli *thioredoxin [98]). Formation of the intramolecular disulfide inactivates the enzyme; treatment with either a reducing agent or a reduced thioredoxin can regenerate active enzyme. Unfortunately, the lack of genetics with *A. fulgidus *makes it difficult to see what role this protein does play in hyperthermophiles. Although enzymes for the third and fourth steps in DIP production have not been identified, the last step has been demonstrated with cell extracts and added CDP-inositol and *myo*-inositol (L. Chen and M.F. Roberts, unpublished results). DIP synthesis required the presence of Mg^2+^.

### E. α-Mannosylglycerate (α MG)

**Figure 9:**
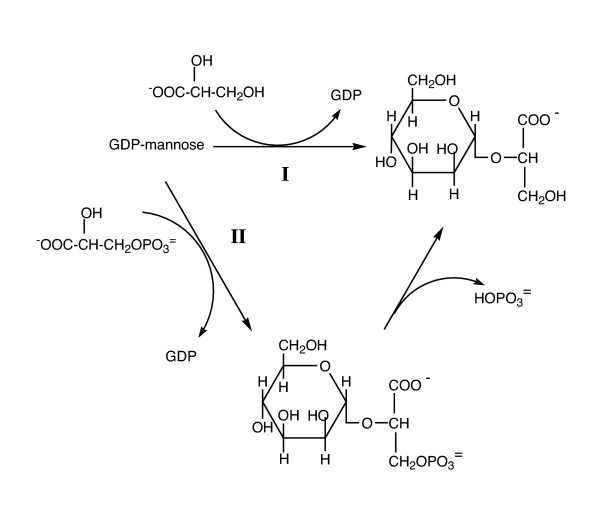
Two pathways exist for α-mannosylglycerate biosynthesis. In (I) GDP-mannose is directly converted to mannosylglycerate. In (II), the GDP-mannose condenses with 3-phosphoglycerate to for mannosyl-3-phosphoglycerate, which is subsequently dephosphorylated to form mannosylglycerate.

The synthesis of this osmolyte has been examined in several hyperthermophiles. There appear to be two distinct pathways (Figure 9). In *R. marinus*, there is a direct condensation of GDP-mannose and D-glycerate to form α MG catalyzed by mannosylglycerate synthase [99]. A second pathway, used by hyperthermophilic archaea, converts GDP-mannose and D-3-phosphoglycerate to mannosyl-3-phosphoglycerate via mannosyl-3-phosphoglycerate synthase, followed by phosphoglycerate phosphatase activity to remove the phosphate group [100,101].

### F. Cyclic-2,3-diphosphoglycerate (cDPG)

**Figure 10:**
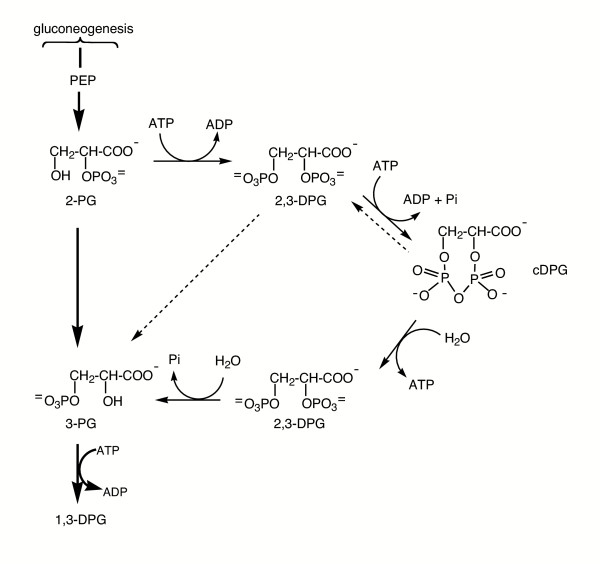
Proposed biosynthesis of cDPG as a pathway linked to gluconeogenesis through 2-PG and 3-PG. The dashed lines indicate the reversible cDPGS reaction of *Methanothermus fervidus*. The solid lines show cDPG and 2,3-DPG interconversions and illustrate the irreversible nature of the cDPGS in *Methanobacter thermoautotrophicus*.

The biosynthesis of the solute cDPG diverts resources from gluconeogenesis by phosphorylation of 2-phosphoglycerate (2-PG) with ATP to form 2,3-diphosphoglycerate (DPG) via 2-phosphoglycerate kinase [102]. Different transformations involving cDPG production and hydrolysis are shown in Figure 10. A novel enzyme, cyclic 2,3-diphosphoglycerate synthetase (cDPGS) [103,104] then converts DPG to the cyclic form with ATP hydrolysis driving the reaction. Hydrolysis of cDPG to 3-PG would shuttle carbon and phosphate back into gluconeogenesis. In *Methanothermus fervidus *cDPGS is reversible, although it prefers the direction of cDPG synthesis [104]. The ability to generate ATP from ADP, inorganic phosphate Pi) and cDPG under these conditions could argue for a role in energy storage in that organism. However, in *Methanobacter thermoautotrophicus*, the K^+^-activated cDGPS appears irreversible (and membrane-bound), suggesting it may have a different role [105]. In soluble cell extracts, hydrolysis of cDPG in the presence of ADP and Pi could not generate ATP [106]. However, a membrane bound hydrolase that is inhibited by K^+ ^and Pi has also been identified [105]. The regulation of cDPG degradation in *M. thermautotrophicus *is consistent with it playing a role as a carbon and phosphate storage compound, although its high concentration in cells clearly indicates it contributes to osmotic balance.

## Transport of Osmolytes

Osmolyte transporters also play important roles in the osmotic response. Some of these transporters are very specific and serve to retrieve any solute released by cells. Others have evolved to scavenge solute or osmolyte precursors so that the more wasteful biosynthetic resources of the cell are not used. Recent years have witnessed progress in identifying and characterizing the proteins responsible for uptake of the osmolytes betaine and ectoine from the medium. In other cases putative transporter genes have been identified but no accumulation of the solute is observed. A summary of the different types of betaine (and one ectoine) transporters is presented in Table 1. Another category of membrane proteins involved in osmolyte movement are the mechanosensitive channels. These are the major players in responding to hypoosmotic stress in that they serve as the conduits for solute removal from the cytoplasm. A recent review on many of the membrane proteins acting as osmosensors can be found in [107].

**Table 1:** Halotolerant or halophilic microorganisms that can transport betaine or ectoinse from the medium.

Solute & Organism	Comments	Reference
**Betaine:**		

*Acinetobacter *sp. F-2-12	in 20% NaCl, cells accumulate 1.26 M betaine and 0.36 M glutamate	[16]
*Actinopolyspora halophila*	cells can synthesize it de novo (oxidation of choline) as well as transport it from the medium	[36]
*Alcaligenes *sp. F-5-7	~1 M betaine when cells grown in complex medium in 20% NaCl	[16]
*Alteromonas *sp. A-387		[16]
*Chromohalobacter israelensis*	betaine in the medium suppresses ectoine biosynthesis	[26]
*Chromobacterium marismortui *A-65	in 20% NaCl, cells accumulate 0.5 M betaine and 0.10 M glutamate	[16]
*Corynebacterium glutamicum*	has genes for four uptake systems including high affinity BetP and a low capacity osmoregulated permease	[111]
*Desulfovibrio halophilus*	1 mM external betaine suppresses sucrose synthesis	[37]
*Listeria monocytogenes*	halotolerant organism also accumulates acetylcarnitine, carnitine, γ-butyrobetaine and 3-dimethylsulfoniopropionate	[110]
*Marinococcus halophilus*	BCCT family transporter BetM	[109]
*Methanohalophilus portucalensis *FDF1	accumulation of external betaine suppresses synthesis of osmolytes; *bta *gene responsible for ABC transporter activated by heat and salt stress	[8]
*Methanosarcina mazei *Gö1	*ota *gene responds to salt shock	[113]
*Methanosarcina thermophila *TM-1	High affinity ABC transporter	[112]
*Nesterenkonia halobia *CCM 2591	in 20% NaCl, betaine is 1.10 M while glutamate is 0.05 M	[16]
*Pseudomonas *sp. F-12-1		[16]
*Tetragenococcus halophilus*	single component transporter (ButA) that is a member of BCCT family; specific for betaine	[108]
*Salinivibrio costicola *A-514		[16]

**Ectoine:**		

*Halomonas elongata*	transporter similar to tripartite ATP-independent periplasmic transporter family (TRAP-T)	[116]
*Marinococcus halophilus*	role likely to be recovery of leaked ectoine	[109]
*Sinorhizobium meliloti*	ABC ectoine transporter identified	[117]

### A. Betaine

Betaine transport is common to a wide variety of halotolerant and halophilic organisms, both bacteria and archaea. There are basically two superfamilies of betaine transporters: (i) secondary transporters that use either the proton motive force or sodium motive force to drive betaine accumulation, and (ii) ATP binding cassette (ABC) transporters that couple ATP hydrolysis to uptake.

Most organisms that internalize this solute do so via a member of betaine choline carnitine transporter (BCCT) family of secondary transporters [108]. The transporter can be quite specific for betaine as in the moderate halophilic lactic acid bacterium *Tetragenococcus halophilus *[108] or Gram-positive *Marinococcus halophilus *[109]. Alternatively, the transporters available can internalize a wider range of solutes. For example, *Listeria monocytogenes *internalizes acetylcarnitine, carnitine, γ-butyrobetaine and 3-dimethylsulfoniopropionate as well as betaine, and the uptake increases the growth rate [110].

Other secondary transport systems have also been described. *Corynebacterium glutamicum *has the usual high affinity BetP uptake system. However, if this and the genes for three other compatible solute uptake systems are deleted, betaine can still be internalized, although the uptake is significantly reduced. The gene identified for the low capacity osmoregulated permease (*lcoP*) codes for a protein (LcoP) resembling a member of the BCCT-family [111]. External osmolarity regulates expression and activity of LcoP.

Methanogens that can transport betaine into the cell tend to use a high affinity transporter [112] that is an ABC transporter. ABC betaine transporters have a nucleotide binding domain that hydrolyzes ATP, a membrane spanning domain, and a substrate binding domain (and/or a periplasmic or extracellular binding protein with a high affinity for betaine). The *ota *(osmoprotectant transporter A) gene of *Methanosarcina mazei *responds to salt shock [113]. *Methanohalophilus portucalensis *FDF1 can transport betaine into the cell as well as synthesize it de novo [8,82]. The *bta *gene responsible for betaine transport in this organism is also an ABC-transporter and is activated by heat as well as salt stress [114]. It is highly specific for betaine – choline, proline and dimethylglycine, and carnitine could not compete with betaine uptake. Interestingly, addition of exogenous betaine or its biosynthetic intermediates induced *bta *expression immediately. The energy required for synthesis of betaine is 36 ATP whereas only two ATP are required for betaine transport by *bta *(S.-C. Chen and M.-C. Lai, unpublished results).

How does betaine finds the transporter? In hyperthermophiles, the high affinity ligand-binding protein ProX serves to bring the betaine to the transporter. Crystal structures of the *A. fulgidus *ProX in the absence and presence of betaine have identified cation-π interactions and non-classical hydrogen bonds between protein and ligand [114]. Similar ligand binding domains have been identified in ORFs in the genomes of other archaea.

### B. Ectoine

Ectoine that is provided in the medium can be internalized by some microorganisms. Growth of halotolerant *Brevibacterium *sp. JCM 6894 is stimulated by exogenous ectoine or hydroxyectoine [115]. In *H. elongata *the transporter for ectoine and hydroxyectoine (TeaA, TeaB, TeaC) is similar to members of the tripartite ATP-independent periplasmic transporter family (TRAP-T) [116]. The K_s_(ect) is 21.7 μM, indicating a high affinity for external ectoine. The role of this transporter appears to be recovery of ectoine leaked from the cell. *Marinococcus halophilus *also can transport external ectoine. In this cell, the EctM gene product is a BCCT family member [108].

In the same vein, a proteomic analysis of *Sinorhizobium meliloti *in media that was supplemented with ectoine detected increased synthesis of ten proteins, eight of which were identified by MALDI-TOF analysis of peptides from the two-dimensional gels [117]. Five of these belong to the same gene cluster (localized on the pSymB megaplasmid), whose components code for the ATP-binding cassette transporter ehu (ectoine/hydroxyectoine uptake). Another cluster of genes (*eutABCDE*) would produce proteins capable of ectoine catabolism. The net result of exposing *S. meliloti *to ectoine is to enhance the production of proteins to internalize and use any of these molecules that escape the cell.

### C. Homologues of Transporter Genes

*Halobacterium salinarum *has two ORFs upstream of transducer genes with significant homology to binding proteins for amino acids and compatible solutes [118]. Deletion mutants indicate that the CoSB/CosT binding/transducer pair, in which the CosB is a membrane-anchored receptor, is critical for chemotaxis towards compatible solutes (in this case betaine). Whether or not the organism accumulates large amounts of betaine (which seems not to occur in *Halobacterium *NRC-1 [119]), this protein pair could function as a chemotaxis signaling pathway for organic osmolytes.

### D. K^+^

A variety of K^+ ^channels have been identified in microorganisms. Structures of various K^+ ^channels, initially a closed, small bacterial channel [120] and more recently a gated K^+^-channel (MthK) from *Methanobacter thermoautotrophicus *[121], have contributed to understanding how these proteins are arranged in membranes. However, these K^+^-channels do not respond to altered osmotic pressure. Rather, different protein complexes appear to regulate intracellular K^+ ^in response to osmotic stress. *H. elongata *uses K^+^-glutamate as an osmolyte. Recent work has identified three genes required for K^+ ^uptake: *trkA*, *trkH*, and *trkI *[122]. The protein expressed by trkA would be analogous to the cytoplasmic NAD^+^/NADH binding protein TrkA in *E. coli *that is required for K^+ ^uptake by the Trk system, while the *H. elongata *TrkH and TrkI are likely to be transmembrane proteins. Experiments with *H. elongata *indicate the TrkI is the main K^+^-transporter in this organism. Similar uptake systems may exist in other halophiles as well.

### E. Membrane Osmosensors

Cells will swell upon hypoosmotic shock as water rushes into the cell. To return to the original cell volume, cells need a rapid means of cytoplasmic solute efflux. All microorganisms have families of gated transmembrane channels that open for solute release when the lateral pressure of the membrane drops below a critical value (for review see [123]). Mechanosensitive channels (Msc) are gated by membrane tension and thought to be primary biosensors for osmoregulation in bacteria [124,125]. Their major role appears to be the rapid and non-discriminating release of solutes upon hypoosmotic shock [125]. Msc fall into three classes: MscL, a pentamer with no solute preference that has large conducting activity; MscS, a heptamer that has smaller conductance, is sensitive to membrane tension, and can exhibit selectivity for anions or cations; MscK, likely a heptamer like MscS that is activated by cytoplasmic K^+ ^[123]. These channels are usually closed but upon changes in membrane tension can open to allow solute efflux. Other osmosensors include the Volume-activated channels (VAC). These have been suggested to respond to hypoosmotic response as anion channels [126]. VAC sensors appear to be responsible for the expulsion of a variety of osmolytes, notably amino acids and polyols.

## Macromolecule Stabilization By Osmolytes – Theories

Along with balancing external osmotic pressure, compatible solutes have also been shown to stabilize macromolecules. There are many theories regarding protein-solute interactions. These can be classified into two types: (i) those that postulate direct solute-macromolecule interactions and (ii) those that hypothesize that macromolecular stability is mediated by solute-induced changes in water structure.

### A. Solute-Macromolecule Interactions: Preferential Solute Exclusion andHydration

**Figure 11:**
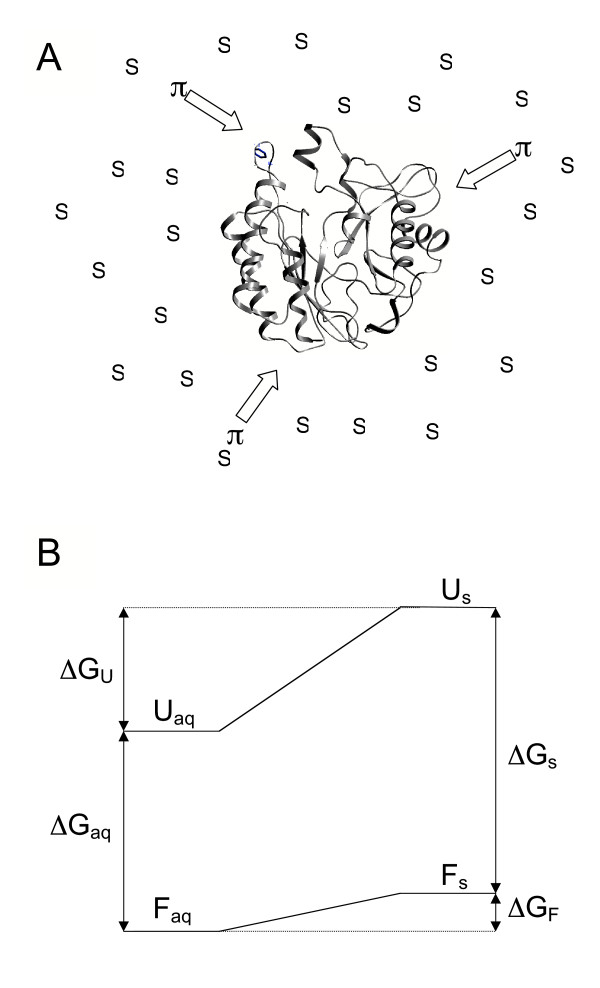
(A) Exclusion of solutes from the surface of a protein increases the concentration of solute in the bulk solution, which in turn increases water surface tension generating osmotic pressure that drives a protein to retain a more compact structure. (B) Osmolytes stabilize proteins to thermal denaturation by differentially raising the energy level of the unfolded state: U, unfolded state; F, folded state; aq, aqueous solution; S, solution containing osmolytes.

Osmolytes in high concentrations compete with water molecules for interactions with protein surfaces. However, it has been proposed that these organic solutes are preferentially excluded from the surface of proteins [127-130]. This in turn leads to preferential hydration of the protein. The increased osmotic pressure generated by the solutes should favor compact folded proteins, which expose less surface area than denatured protein (Figure 11A). The size of internal cavities and internal water should be reduced as well [131]. Differential interactions of organic solutes with folded and denatured proteins also contribute to their stabilization effects. Bolen and coworkers [132] have proposed that, compared to water, solutes have more unfavorable interactions with the peptide backbone and since unfolded protein has more available backbone, this biases the equilibrium to a folded protein (Figure 11B). This would suggest that osmolytes that impart stability actually interact with the unfolded state of the protein, shifting the equilibrium to promote the folded configuration (the 'osmophobic effect' [132]). The free energy of the denatured state is higher than that of the native state, making population of this state energetically unfavorable (Figure 11B). Any interactions of osmolytes with hydrophobic residues of the unfolded protein do not overcome the osmophobic effect, nor do they interfere with the hydrophobic effect.

### B. Solute-Induced Changes in Water Structure

Ionic solutes will have a pronounced effect on water structure and these interactions will affect macromolecule stability. Neutral salts do not have the same effect on structure and solubilities of proteins. The Hofmeister series of ions reflects the ability of different ions to bind water [133,134]. 'Kosmotropes' (order makers) exhibit a strong interaction with water, while 'chaotropes' (disorder makers) exhibit weaker interactions with water than water has with itself. It is thought that kosmotropes bind water strongly and aid in preserving the hydration layer around the macromolecules. These solutes prefer interactions with water rather than the protein surface, hence preserve preferential hydration of the protein. Chaotropes can displace water from the protein surface and contribute to destabilization of structure by dehydrating the macromolecule. Many osmolytes are strikingly similar to the ions of the Hofmeister series: amino acids resemble ammonium acetate and the methylamines are functionally similar to quarternary ammonium ions [135]. Their effects on proteins should then be similar to those in the Hofmeister series.

Collins and coworkers have shown that the Hofmeister series is actually a function of the apparent dynamic hydration number of the ion [136,137], with the more hydrated an ion, the greater the stabilization of macromolecules. The calculated hydrated radius for each ion nicely coincides with the ionic strength. Collins postulated that the effect of an osmolyte on another solute (in this case the macromolecule) depends on the extent it perturbs the solvation layer of the other solute. If the osmolyte is tightly hydrated, it cannot as easily interact with the macromolecule solvation layer, an event that would destabilize the macromolecule.

Other considerations of water structure and the influence of solutes on charged regions of macromolecules have suggested that the water layer at the protein surface is more dense and reactive than bulk water [138]. Patches of dense water, along with counterions, would cover charged surface regions of the protein, while inert zones of low-density water would be found around hydrophobic groups. Macromolecular crowding (see below) would also influence this. In dilute solutions, changing the density of the bulk water would be energetically unfavorable because of the large relative volume. When the solution is concentrated (a cell typically has 2 to 4 g water/g dry weight [138]), the volume of surface water becomes comparable to the volume of bulk water, allowing density changes to occur.

### C. Osmolytes, Excluded Volume, and Pressure Effects

The crowded and inhomogeneous environment of the cell also contributes to stabilization of folded proteins [139-141]. The presence of solutes such as osmolytes, 'macrosolutes' such as cofactors, and other macromolecules aid in stabilizing proteins by decreasing the accessible volume, shifting the equilibrium between the folded and unfolded state of proteins to favor the more compact folded state.

This has relevance to pressure stress as well. Increasing hydrostatic pressure should promote water penetration from protein surface to the core. With osmolytes that are bigger than water, this penetration is less likely if critical water is around the protein and bulk water is diluted with the osmolyte. Studies of *Photobacterium profundum *solutes at 1 and 280 atm are perhaps the best evidence that small solutes can repel / inhibit water penetration at high hydrostatic pressures [52]. Elevated hydrostatic pressure tends to denature proteins [142], presumably by enhancing water penetration into the protein core [143]. In this deep sea organism, β-hydroxybutyrates accumulate to high intracellular levels at 280 atm. Perhaps these negatively charged solutes aid in preventing water penetration, both with an excluded volume effect but also by altering water structure in the vicinity of the proteins.

The observation of osmolyte cocktails in different types of cells likely reflects selection of solutes that cover different aspects of these effects – destabilization of the denatured state, retardation of water penetration in protein cores, and optimal modulation of water density for a particular cytoplasm. For example, one might expect thermophiles to have different solutes than mesophiles if solute exclusion is less important than other aspects of osmolyte effects (perhaps at high temperatures altering water structure is more important). The accumulation of multiple solutes may be explained by slightly different (and potentially overlapping) effects for each specific solute. For example, in a cell with multiple zwitterions, perhaps solute size or charge distribution are important in stabilizing water at some protein surfaces, while for other proteins preferential exclusion is the dominant effect. In cells with mixed zwitterions and anions, perhaps the anions are better excluded by proteins with a net negative charge, but if the organic anions are balanced by intracellular K^+ ^there may be a limit on their intracellular concentration and so zwitterions are also accumulated.

## Experimental Effects Of Osmolytes On Macromolecules

### A. Thermoprotection of Proteins

Heat stress often provokes similar responses to salt stress. Organisms adapt to high external salinity by accumulating osmolytes, and the same solutes accumulated in vivo can also affect stability of microorganisms to thermal stress. *E. coli *cells adapted to grow in high salt contain increased betaine. Diamant et al. [144] showed that heat shock of these salt-adapted cells dramatically reduces the protein aggregation seen in non-adapted cells under the same stress. This behavior was suggested to result from osmolyte (specifically betaine, glycerol, proline or trehalose) activation of chaperones GroEL, DnaK, and ClpB. While such interactions could be shown at low osmolyte levels, at high osmolyte levels, refolding of proteins was reduced, possibly because of specific deleterious interactions of the osmolytes with chaperones.

In many cases, these small molecules assist in protein stabilization and/or refolding in vitro. The in vitro studies with purified enzymes allow one to explore any protective effects or unusual behavior of novel compatible solutes. These studies are consistent with the hypothesis that osmolytes are selected for their unfavorable interactions with peptide backbones [132].

Rabbit muscle lactate dehydrogenase has been used to test the effect of ectoine, hydroxyectroine, and their biosynthetic precursors DA and NADA on thermostability of this enzyme. At 55°C NADA enhances thermostability as measured by protection of the enzyme from thermal inactivation [89]. Hydroxyectoine is more effective than ectoine and NADA at stabilizing proteins to heat stress. The one real difference with hydroxyectoine is a hydroxyl group on the ring. Perhaps this further functionalization of the (now trihydro)pyrimidine ring aids in organizing water and maintaining high surface tension at high temperatures.

Ribonuclease has been a popular target for osmolyte stabilization studies. This disulfide crosslinked enzyme can be reversibly unfolded in the absence of reducing agents. The effects of some of the more exotic osmolytes have been examined with this enzyme. 2-O-α-Mannosylglycerate, 0.5 M, increases the mid-point of the thermal denaturation curve, T_m_, by 7°C as well as increases the heat capacity for the protein [145], an effect consistent with the solute destabilizing the denatured state with respect to folded protein. Other studies with ribonuclease D show that a variety of zwitterionic osmolytes dramatically increase T_m _for the protein. As an example, 6 M sarcosine increases T_m _by 24.6° at pH 5 [146]. Crystal structures of the ribonuclease fail to detect any bound osmolyte or alterations in water bound to the protein. The data support the hypothesis that osmolytes stabilize proteins by perturbing unfolded states, which biases the equilibrium to a compact, folded state.

The charge distribution of an osmolyte can be important to its biological activity as well. Solutes used by bacteria and archaea have not been examined since the pKa of any functional groups are well outside accessible pH ranges for maintaining native proteins. Trimethylamine N-oxide (TMAO), a common solute in eukaryotes, is zwitterionic above pH 6 (pKa of 4.66), and it is this state of the molecule that is critical for its stabilization of proteins [147]. At low pH it no longer acts as a good thermoprotectant. While this observation may not have physiological relevance, it aids in our understanding of osmolyte properties important for their biological effects. TMAO has been shown to decrease the entropy of the unfolded state of onconase through a solvophobic effect [148]. This solute clearly diminishes the unfolding rate while having little effect on the stability of the native protein. For onconase, TMAO appears to induce a local structural change that retards unfolding.

The solute exclusion theory would argue for little specificity in osmolyte effects on macromolecules. However, there are many studies that clearly show preferential stabilization by solutes. Osmolytes can counteract denaturants such as urea. Potassium D- or L-glutamate (0.25 M) counteracts the effect of urea on glutaminyl-tRNA synthetase from *Escherichia coli *by shifting the equilibrium between the native and molten globule and molten globule to unfolded protein to a higher urea concentration [149]. However, for this protein other osmolytes (sorbitol, TMAO, inositol) cannot induce the same shift. A major conclusion of these studies is that the ability of an osmolyte to counteract urea denaturation depends on specific osmolyte-protein interactions.

As another example, two archaeal rubredoxins have been shown to be stabilized to quite different extents by α-diglycerol phosphate [150]. Their structures are similar, except that one is missing a hairpin loop. There are small conformational changes induced by α-DGP (or mannosylglycerate) and evidence for solute inducing a more compact state of the protein, and the occurrence of weak, specific interactions between osmolyte and protein surface.

Osmolytes can affect protein conformation and motions of native structures as well. TMAO induces α-helix formation of alanine-base peptides [151]. Compatible solutes also attenuate structural fluctuations as measured by amide hydrogen-deuterium exchange rates [152-154]. Osmolytes certainly inhibit slow, large unfolding transitions, but they can also modulate fast exchange rates as well [155]. Tryptophan phosphorescence has been used to probe the flexibility of the native structure of azurin and a number of mutants [131]. The sugar dampens fluctuations only for loose internally hydrated macromolecules and those with thermally expanded conformations. The sucrose (and presumably other polyols) will shift the equilibrium of protein conformations to a more compact rigid form.

One of the interesting questions is whether or not solutes in halophiles stabilize proteins in the same manner as for nonhalophiles. An interesting case in point is the effect of KCl on the dihydrofolate rductase (DHFR) from *Haloferax volcanii *compared to that from *E. coli *[156]. The protein from the extreme halophile is much more acidic and one might think the stabilization effects by K^+ ^would differ compared to the mesophilic, non-halophilic homologue. The *H. volcanii *DHFR requires at least 0.5 M KCl to stay folded, while the *E. coli *protein is inactive above 1 M KCl. Yet the effect of salts on the stability of the proteins to urea is similar, if one compares stability at the appropriate physiological ionic strength. This work shows that salts stabilize the DHFRs by a common mechanism – preferential hydration and the Hofmeister effect of salt on the activity and entropy of the aqueous solvent. Although one could imagine hydrated salt networks occurring in the halophilic protein leading to halophile-specific stabilization, that is not the case.

### B. Interaction with Nucleic Acids

Although most research into how osmolytes affect macromolecular stability has concentrated on proteins/enzymes, these solutes also affect nucleic acid stability. The addition of high concentrations of zwitterionic solutes increases the dielectric constant of the solution that, in turn, decreases ionic interactions and affects the DNA duplex. Isolated studies have explored the effect of zwitterionic solutes on nucleic acid stability. For example, betaine has been shown to eliminate the dependence of dsDNA melting on the base pair composition [157] and to enhance amplification of GC-rich templates [158] by lowering the T_m _for the template. High concentrations of compatible solutes also alter accessibility of regions of the DNA to nucleases. Malin et al. [159] showed that ectoine and hydroxyectoine alter the DNA conformation such that endonucleases can no longer cleave it.

## Biotechnological Applications Of Osmolytes

The properties of osmolytes make them suitable for a variety of uses in biotechnology as long as one can generate reasonable quantities either in vivo or in vitro. Induction of osmolytes in cells can increase protein folding, so that engineering osmolyte biosynthesis genes in an organism should improve its salt tolerance. The trick is to couple osmolyte production to salt stress. For in vitro uses, large amounts of pure solutes are needed. In many cases, the solutes can be supplied by 'bacterial milking.' Both ectoine and hydroxyectoine have been produced in large quantities using *Halomonas elongata *[160]. Bacteria in high NaCl are transferred to low osmolarity medium where they excrete the now excess solutes. Re-exposure of the bacteria to high salt induces them to re-synthesize the osmolytes. Repeated transfers between low and high osmolarity media should dramatically enrich the media in the osmolytes. Purification of the solutes then relies on chromatographic steps. This process is the basis of the German biotechnology company Bitop http://www.bitop.de/sources/html/e/index.htm that has developed preparative methods for many of the unique osmolytes produced by microorganisms.

### A. Chemical Chaperones for Protein Folding

Insoluble or misfolded overexpressed proteins can often be partially denatured and refolded in the presence of osmolytes. A specific example is the use of osmolytes to enhance the yield of folded, functional cytotoxic proteins directed to the periplasm of *E. coli *[161]. Cells grown in 4% NaCl with 0.5 M sorbitol and supplemented with 10 mM betaine can accumulate large amounts of the target protein in the periplasm (this was tried with immunotoxins). Protein is released by freeze-thaw cycles. Both high osmotic strength and added compatible solutes (in this case betaine and sorbitol) are necessary for high yields of protein.

In the same vein, ectoine, betaine, trehalose, and citrulline have been shown to inhibit insulin amyloid formation in vitro [162]. This observation may provide directions for designing small molecules to inhibit myelin formation associated with neurodegenerative disorders.

### B. Enhancing PCR

Several osmolytes (notably betaine, ectoine) have been shown to be useful in PCR amplification of GC-rich (72.6% GC) DNA templates with a high T_m_. In particular, ectoine was shown to outperform regular PCR enhancers; it works by reducing the DNA T_m _[163]. Interestingly, hydroxyectoine increases the T_m _of duplex DNA. However, the optimal solute for these experiments is homoectoine (4,5,6,7-tetrahydro-2-methyl-1H-[1,3]-diazepine-4-carnoic acid), a synthetic derivative of ectoine with the ring expanded by one carbon. For betaine the effective range of solute is 0.5 to 2.0 M; for ectoine much less (0.25 to 0.5 M) is needed for the same effect. It would be intriguing to see what effect DIP type solutes have on PCR since they are synthesized by hyperthermophiles above 80°C.

### C. Cryo-protection of microorganisms

Organic osmolytes have also been used as cryo-protectants. In a recent study, the ability of betaine to act as a cryo-protectant during freezing of diverse bacteria was examined. Betaine is often much better than two common cryo-protectant mixtures, serum albumin and trehalose/dextran, particularly under conditions simulating long-term storage [164]. It is better than the other treatments at preserving long term viability for microorganisms like *Neisseria gonorrhoeae *and *Streptococcus pneumoniae*. Betaine is as effective as glycerol for liquid nitrogen freezing of halophilic archaea, and neutrophilic Fe-oxidizing bacteria.

### D. Use in cosmeceuticals and pharmaceuticals

The ability of osmolytes to aid in protecting cells from diverse stresses has led to the use of at least one of them, ectoine, in the cosmeceutical industry. Ectoine has been shown to protect skin from UVA-induced cell damage [65]. Based on this, RonaCare™ Ectoin, produced by Merck KgaA, Darmstadt, is presently in use as a moisturizer in cosmetics and skin care products.

Osmolytes have not been developed as reagents in the pharmaceutical industry, in part because as 'compatible solutes' they interact minimally with cellular machinery. However, their ability to stabilize biomolecules may have some very specific uses. As an example, the German company Bitop in collaboration with researchers at the Cologne University Clinic is exploring the use of these solutes in certain cancer therapies where they may protect tissues against vascular leak syndrome, a severe side effect of anti-caner agents.

### E. Generation of Stress-Resistant Transgenic Organisms

Insertion of genes for osmolytes into non-halotolerant organisms should increase their ability to withstand salt stress. Plants are a good target for these types of experiments since they are often exposed to drought conditions that would concentrate salt. A few reports of transgenic plants suggest that eventually this strategy might be useful. *Arabidopsis thaliana *transformed with a choline oxidase gene (which is needed to synthesize betaine) from *Arthrobacter globiformis *has a significantly improved tolerance of salt stress along with improved cold and heat tolerance [166]. Transgenic tobacco with *E. coli betA *and *bet B *genes has also been constructed. This modified plant exhibits better salt and cold tolerance [167]. Inserting the *H. elongata ectABC *genes also confers hyperosmotic tolerance on cultured tobacco cells [168]. This was shown to increase the hyperosmotic tolerance of cultured cells, although only a small amount of ectoine accumulated. Other recent work to introduce genes for synthesizing osmolytes in plants [169] as a way to improve stress tolerance has, so far, not led to high accumulation of the osmolytes. Further developments await a determination of what limits osmolyte levels in plant cells.

## Conclusion

Clearly, there are many different organic solutes used for osmotic balance in halotolerant and halophilic microorganisms [170]. Except for α-glutamate, the solutes that are accumulated are not intermediates in biochemical pathways. They are appropriately modified so that they are not chemically reactive even if they occur at high concentrations (i.e., no reactive groups in carbohydrates) and would have little affinity for the macromolecules that interact with precursors. In most cases these organic solutes are not metabolized by the cells that accumulate them. With these properties, they nicely fulfill Brown's original definition of a compatible solute. However, some of the more uncommon solutes raise interesting questions. For example, what is it about DIP that makes it a solute of choice in hyperthermohiles growing above 80°C?

One of the recurrent themes is that most microorganisms use a cocktail of solutes unless an external solute such as betaine is provided. One solute may be the major species, but there are usually several solutes at moderate concentrations in a cell, and the balance among the solutes can be modulated by growth stage and carbon and nitrogen substrates. In several of the cases described, there is a switch from one type of solute to another with increasing external NaCl. The most common change is from anionic organic solutes to neutral or zwitterionic solutes (e.g. glutamates to Nε-acetyl-β-lysine in several methanogens, α-mannosylglycerate to α-mannosylglyceramide in *Rhodothermus marinus*). This suggests that internal cation concentrations are intimately linked with the osmolyte pools. There is also evidence that when challenged with increased external NaCl, many organisms exhibit an initial response, which in terms of solutes may include K^+ ^internalization and α-glutamate synthesis. The solutes in this first response are then replaced by a steady-state of other solutes reflecting the adapted cell osmolyte composition. How the cell coordinates this is an area that certainly needs to be explored.

In the past decade there has been significant progress in defining biosynthetic pathways and in identifying the enzyme components for many of these solutes. Ectoine, betaine, and mannosylglycerate synthesis have been examined in detail in several cells. Recent work has at least identified the genes coding for the lysine aminomutase and acetyltransferase needed for Nε-acetyl-β-lysine biosynthesis, and several of the enzymes along the biosynthetic pathways for β-glutamate and DIP have been characterized. Yet the enzymes responsible for the synthesis of other solutes (notably the neutral dipeptide and derivatized amino acid, α-diglycerol phosphate, and sulfotrehalose) have not been explored. In order to understand how external NaCl concentrations are linked to osmolyte synthesis (or removal) such information is critical.

Significant work has also been carried out to understand the thermoprotective features of osmolytes. It has become clear that solutes can have specific effects on protein dynamics and appear to limit some types of motions with the net result of stabilizing folded rather than unfolded structures. As more examples appear we should have a better idea of some of the unusual properties of some osmolytes. For example, modified polyols and carbohydrates are often used by cells that grow at high temperatures as well as high salt. Do these solutes alter solvent structure in a uniform way? Are they better than other solutes in aiding as chemical chaperones for protein folding/refolding?

Regardless of the details of osmolyte biosynthesis and interactions, it is clear that there is a use for these solutes in the biotechnology arena. Stabilization of proteins or enhancing PCR is an obvious application of these solutes, and only the most common solutes have been tried thus far. One might even improve upon the natural solutes with synthetic molecules if one has a firm grasp of how they affect different systems. Some of the more unusual solutes may have particularly interesting properties that could be exploited either in vitro or in vivo. Engineering foreign osmolyte pathways into other cells has not been very successful but such transgenic organisms will certainly be optimized in the future. It will certainly be interesting to see what new information is provided in the next 5 years or so.

## Competing interests

The author(s) declare that they have no competing interests.
